# Structure and Properties of Biodegradable Poly (Xylitol Sebacate-Co-Butylene Sebacate) Copolyester

**DOI:** 10.3390/molecules25071541

**Published:** 2020-03-28

**Authors:** Marta Piątek-Hnat, Kuba Bomba, Jakub Pęksiński

**Affiliations:** 1 West Pomeranian University of Technology, Szczecin, Science, Piastów Ave. 17, 70-310 Szczecin, Poland; bk34688@zut.edu.pl (K.B.); jakub.peksinski@zut.edu.pl (J.P.); 2 Faculty of Chemical Technology and Engineering Piastów Ave. 42, 71-065 Szczecin, Poland; 3 Faculty of Electrical Engineering, Sikorskiego Ave. 37, 71-313 Szczecin, Poland

**Keywords:** ester elastomers, cross-linking, chemical structure, sugar alcohols, ^1^H NMR

## Abstract

In this work, a bio-based copolyester with good mechanical properties was synthesized and characterized in terms of structure, main properties and biodegradability Determining the chemical structure of such materials is important to understand their behavior and properties. Performing an extraction of insoluble cross-linked polymer using different solvents allowed us to analyze how the polymer behaves when subjected to different chemical environments, and to obtain soluble samples suitable for more in-depth analysis. Chemical structure of poly (xylitol sebacate-co-butylene sebacate) was determined by a ^1^H NMR and FTIR analysis of both prepolymer gel sample and samples obtained by extraction of cross-linked polymer using different solvents. Block structure of the copolymer was confirmed by both NMR and DSC. Gel fraction, swelling value, water contact angle, and mechanical properties were also analyzed. Biodegradability of this material was confirmed by performing enzymatic and hydrolytic degradation. Synthesizing sugar-alcohol based copolyester using three monomers leads to obtaining a material with interesting chemical structure and desirable mechanical properties comparable to conventional elastomers.

## 1. Introduction

Sugar alcohols as monomers for polymer synthesis have recently received an appreciable amount of attention from researchers, and have been used as substrates to synthesize a wide variety of materials with very different properties and possible applications. These materials include compounds with sugar alcohols scaffolds and azo-arms which can be reversibly photochemically liquefied and solidified [1], shape-memory poly (mannitol sebacate)/cellulose nanocrystal composites [2], hydroxyapatite composites with poly (sorbitol sebacate malate) matrix [3] polyurethanes with sorbitol as a chain extender for self-healing materials [4] and protective coatings [5]. Xylitol in particular has been used as a monomer for synthesis of multiple materials with different characteristics, which include: core-shell electrospinnable poly (xylitol sebacate) [6,7], elastomeric copolyester with two dicarboxylic acids used as other monomers [8], and autofluorescent poly (xylitol-dodecandioic acid) [9]. Xylitol was also used as a substrate to develop injectable poly (xylitol-co-maleate-co-PEG) hydrogels [10], and star-shaped lactide and ε-caprolactone oligomers with xylitol and other sugar alcohols as core molecules [11]. Xylitol has also been used to synthesize poly (xylitol sebacate) which was analyzed by ^13^C NMR and determined to be a linear chain comprised mainly of 1-acyl and 1,5-diacyl substitutions. Secondary and tertiary hydroxyl groups have very low reactivity, which lead us to conclude that our polymer has a non-branching linear structure [12].

Possibility to fine-tune thermal and mechanical properties, and degradation times of sugar-alcohol based polyesters was investigated, by performing syntheses with different dicarboxylic acids [13,14], different sugar alcohols [15,16], or by changing the stoichiometric ratio of monomers [16,17,18]. Sugar alcohol based polyesters can also be synthesized using an additional monomer - with dicarboxylic acid, sugar alcohol and a diol as the third monomer. Properties of such copolymers can be modified by changing the chain length of diols used for the synthesis [19]. Such copolyesters can also be synthesized using enzymatic catalysis with Candida antarctica Lipase B as a catalyst [20]. Various biodegradable multiblock copolymers synthesized using dicarboxylic acid and butylene glycol have also been described in the literature [21,22,23].

Taking into account the considerable amount of interest received by sugar alcohols as monomers for polymer synthesis, and our earlier analysis of physiochemical properties of xylitol-containing polymer [24], and sorbitol-containing polymer [25] we have concluded that the next logical step in our research was an exact examination of chemical structure and properties of poly (xylitol sebacate-co-butylene sebacate) copolymer which is a good example of sugar-alcohol-based materials. In order to do so, an extraction on insoluble cross-linked polymer using many different solvents was performed. It allowed us to analyze how the polymer behaves after being subjected to different chemical environments, and to obtain soluble samples suitable for more in-depth analysis.

## 2. Results and Discussion

**Figure 1:**
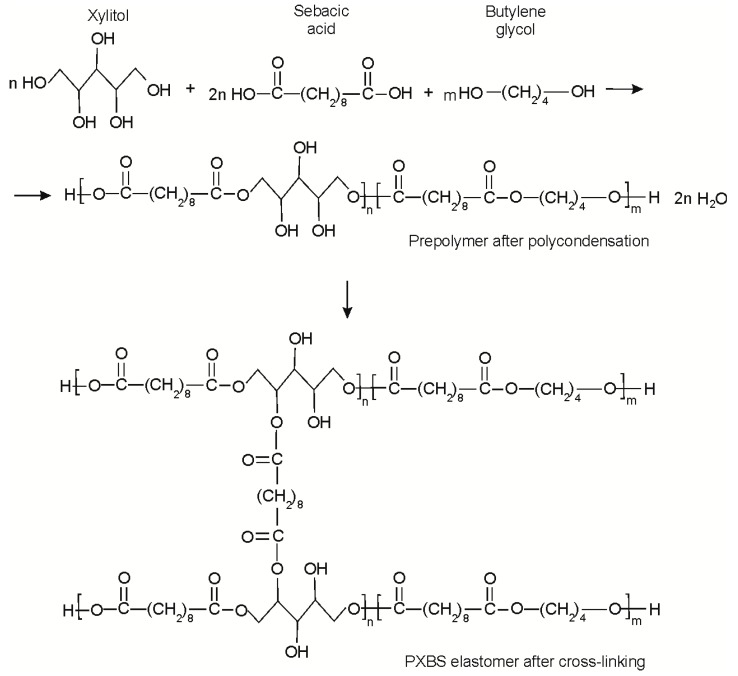
Reaction scheme of poly (xylitol sebacate-co-butylene sebacate) (PXBS) synthesis.

As a result of the synthesis described in 2.1 poly (xylitol sebacate-co-butylene sebacate) (PXBS) was obtained. Reaction scheme is shown in Figure 1. Polymer after cross-linking is characterized by mechanical properties typical for elastomer group (0.93 ± 0.25 MPa stress at break, 306 ± 64% elongation at break) and has hydrophilic (46° contact angle) surface properties.

### 2.1. Swelling

**Figure 2:**
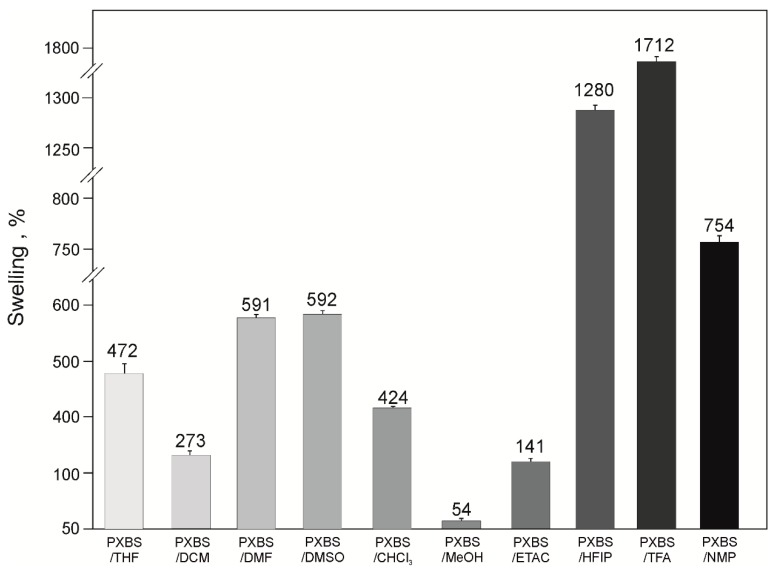
Swelling of PXBS.

Results of the swelling test are shown in the Figure 2. Swelling value in the range of 400–600% can be observed for samples which resided in THF, DMF, DMSO, and CHCl_3._ Sample which resided in MeOH has the lowest swelling value. Highest swelling values are exhibited by samples which resided in HFIP (1280%) and TFA (1712%). Those fluorinated solvents penetrate the polymer network the most and cause its degradation. The degradation of polymer network was confirmed by DSC analysis which shows the lowest change in heat capacity during glass transition for polymer samples which resided in those solvents.

### 2.2. Gel Fraction

**Figure 3:**
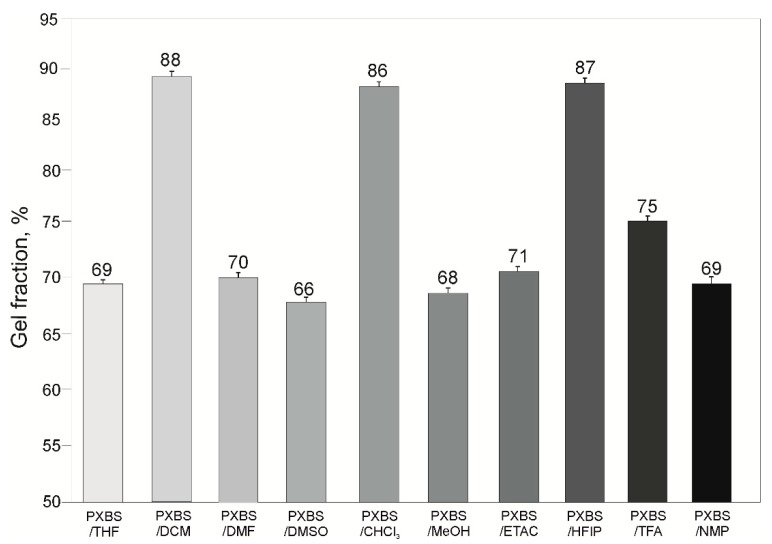
Gel fraction of PXBS.

Values of gel fraction (Figure 3) for all samples are in 65–90% range. Relatively high gel fraction in samples extracted using the most penetrating solvents (TFA and HFIP) is caused by solvent being confined deep within the polymer network which makes it impossible for it to completely evaporate. Solvent most suitable for purification of PXBS is DCM, because it has the least destructive effect on the polymer structure, as confirmed by the highest gel fraction value for the sample extracted by this solvent.

### 2.3. Fourier Transform Infrared Spectroscopy (FTIR)

**Figure 4:**
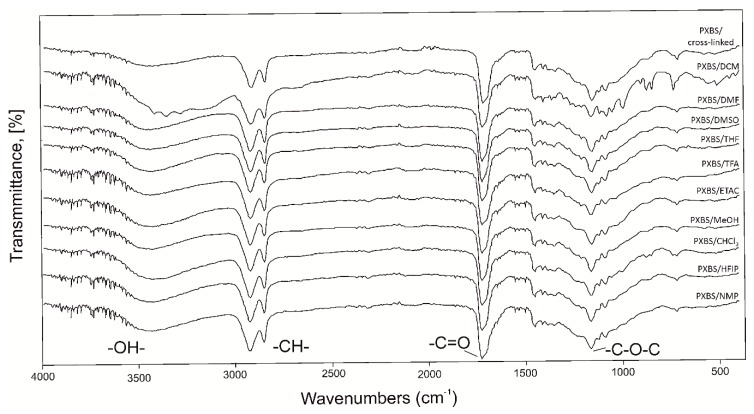
FTIR spectra of PXBS sample after cross-linking and samples of insoluble cross-linked polymer fraction left after extraction (gel fraction).

**Figure 5:**
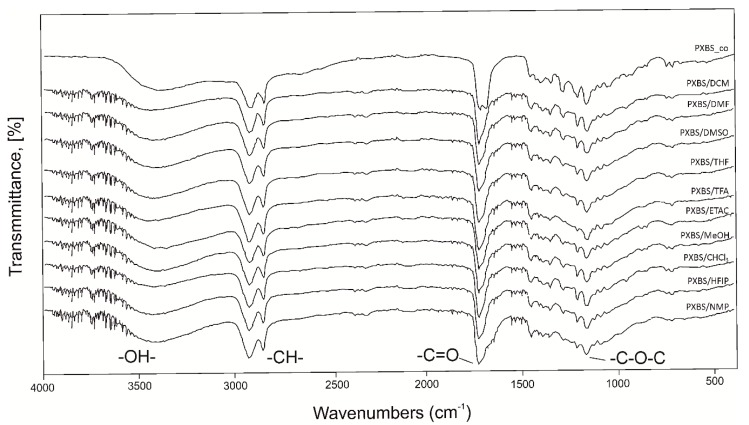
FTIR spectra of PXBS sample after polycondensation and samples of soluble polymer fraction obtained by extraction (sol fraction).

Figure 4 shows FTIR spectra of sample of cross-linked polymer, and samples of insoluble cross-linked polymer fraction left after extraction with various solvents (gel fraction). Figure 5 shows spectra of sample taken directly after polycondensation, and samples of soluble polymer fraction obtained by extraction (sol fraction). FTIR spectra show transmittance peaks typical for a polyester structure. Peak at about 1150 cm^−1^ can be attributed to C-O-C groups, peak at about 1720 cm^−1^ corresponds to C=O groups, peak at about 2900 cm^−1^ can be assigned to alkyl groups, and peak at about 3440 cm^−1^ can be attributed to intermolecularly associated OH groups. For samples after extraction, due to the C-O-C bonds breaking apart during the extraction process there is a slight separation in the peak at about 1700 cm^−1^ because signals from dicarboxylic acid emerge. Another sign of the degradation process is increase in peak intensity in peaks assigned to -OH groups. For insoluble polymer sample after extraction by DCM there is an increase in intensity of non-intermolecularly-associated -OH groups due to the hydrogen bounds breaking apart.

### 2.4. NMR Spectroscopy

**Figure 6:**
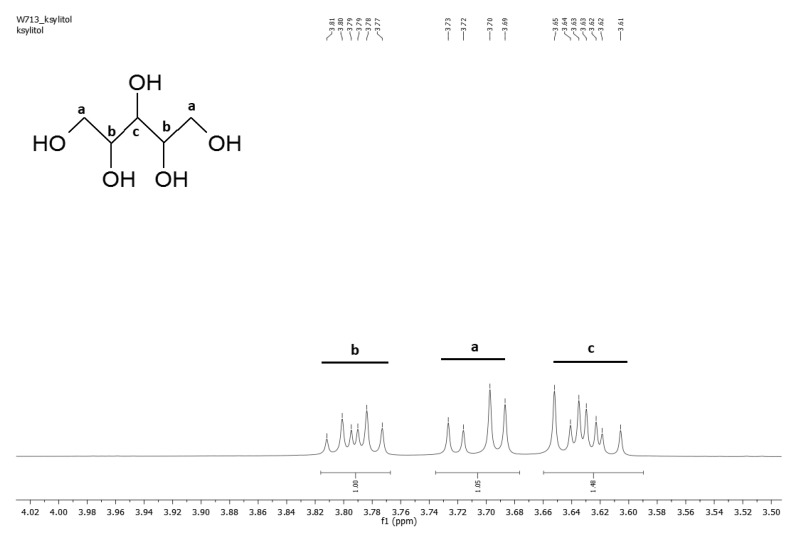
NMR spectrum of xylitol.

**Figure 7:**
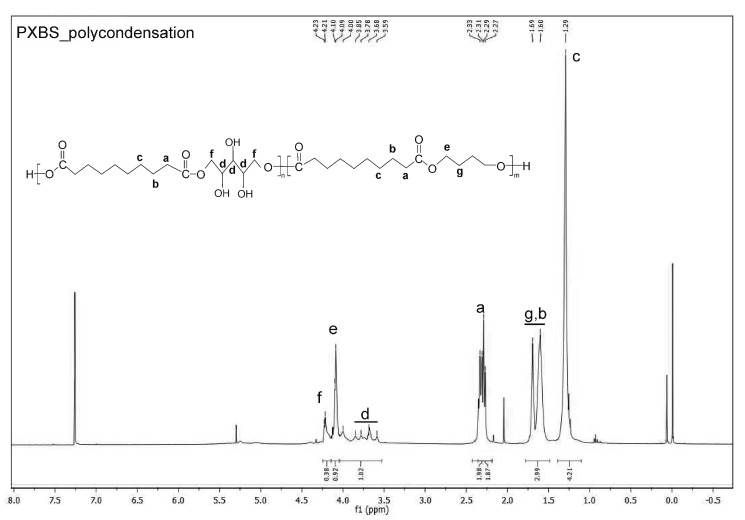
*NMR spectrum of PXBS* after polycondensation.

**Figure 8:**
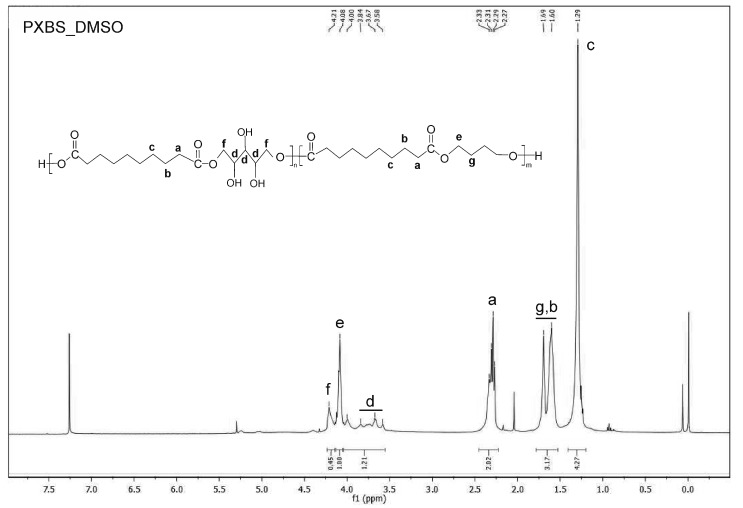
NMR spectrum of sample of soluble PXBS fraction obtained by extraction in DMSO solvent.

**Figure 9:**
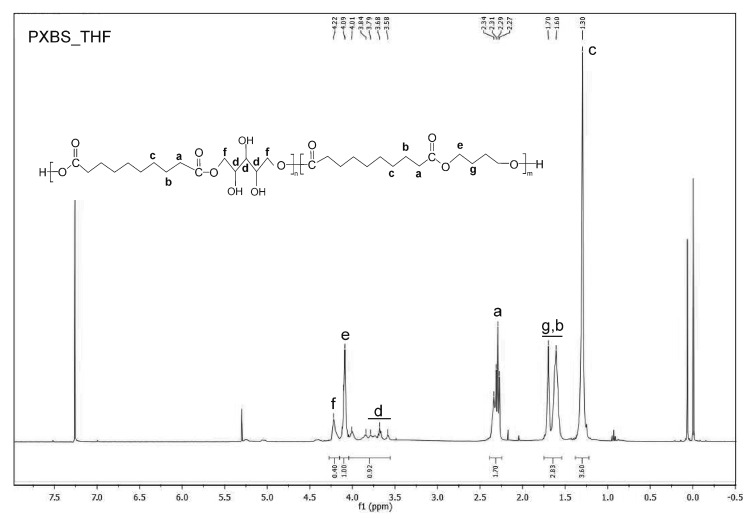
NMR spectrum of sample of soluble PXBS fraction obtained by extraction in THF solvent.

**Figure 10:**
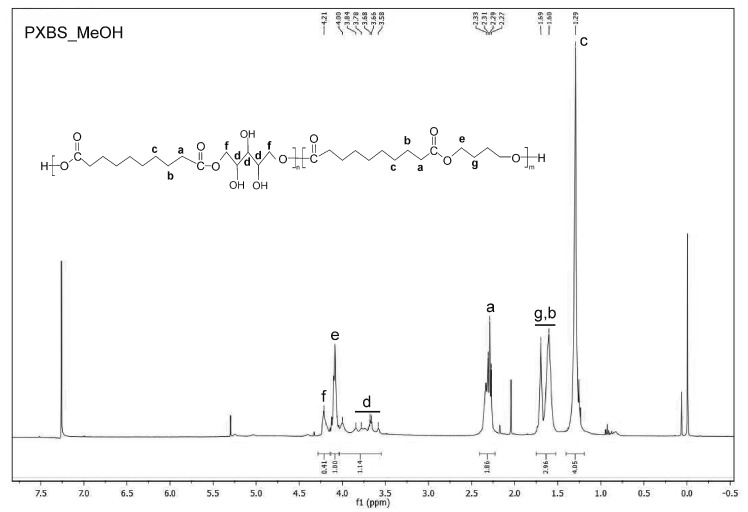
NMR spectrum of sample of soluble PXBS fraction obtained by extraction in MeOH solvent.

^1^H NMR analysis was performed in order to confirm if theoretically assumed structure of the PXBS copolymer was correct. Indeed, the ^1^H NMR measurements (Figure 6, Figure 7, Figure 8, Figure 9 and Figure 10) have proven that the copolymer structure is made up of xylitol-sebacic acid and butylene glycol-sebacic acid segments. Peak at about 4.21 ppm corresponds to the proton adjacent to the oxygen atom which is a part of the ester bond between xylitol and sebacic acid. Peak at about 4.08 ppm was attributed to the proton adjacent to the oxygen atom making up the ester bond between butylene glycol and sebacic acid. Following signals were assigned to alkyl groups in sebacic acid: peak at about 2.30 ppm corresponds to CH_2_(a) group, doublet at 1.60 and 1.70 ppm was attributed to CH_2_ (b), and CH_2_ (g) group, and peak at about 1.30 was ascribed to CH_2_(c) group. Peaks between about 4.00 and 3.58 ppm correspond to CH_2_(d) groups in xylitol. Furthermore, it is worth noting that ^1^H NMR spectrum of sample taken directly after polycondensation, and samples obtained by extraction of the cross-linked material are almost identical, which means that the extraction process completely destroyed the cross-links between polymer chains. The ratio of peak areas from peak at 4.08 ppm to the peak at 4.21 ppm is 2.42, which means that on average, for every xylitol-dicarboxylic acid ester bond there are 2.42 bonds between butylene glycol and dicarboxylic acid. This leads to a conclusion that xylitol is less reactive than butylene glycol in synthesis carried out using three monomers. Considering the fact that not every xylitol particle becomes a part of the linear, non-cross-linked polymer we assume that there are some unreacted xylitol left-overs confined within cross-linked polymer network.

Peak at about 5.4 is an average signal from protons from both carboxyl and hydroxyl groups. This signal averaging is caused by the rapid exchange of protons from both these groups [26]. Presence of this peak is a confirmation that there is some unreacted dicarboxylic acid in the sample taken directly after polycondensation. 

### 2.5. Differential Scanning Calorimetry (DSC).

**Figure 11:**
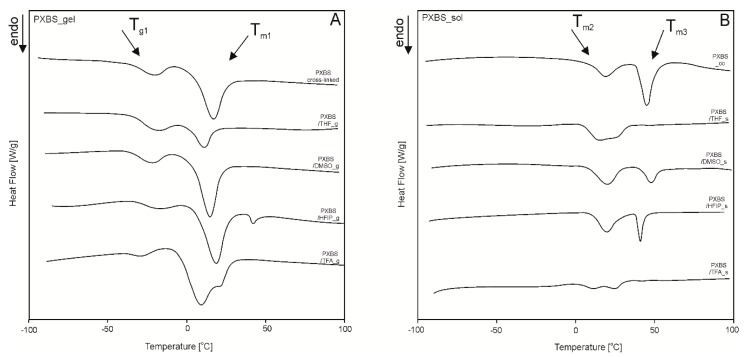
DSC thermograms of PXBS sample after cross-linking and samples of insoluble cross-linked PXBS fraction left after extraction (**A**) and thermograms of PXBS sample after polycondensation and samples of soluble PXBS fraction obtained by extraction (**B**).

In Table 1 (also available in Supplementary Materials) and Figure 11 results of DSC analysis are shown. For the sample of cross-linked material and samples of insoluble cross-linked polymer fraction left after extraction by different solvents (gel fraction) two transition temperatures can be seen. Presence of glass transition temperature (T_g1_) and associated with it change in heat capacity (∆c_p_) is a confirmation of cross-linked amorphous structure of the polymer. Melting transition (T_m1_) is the result of the presence of crystalline areas confined within amorphous polymer network. Extracting the polymer with the most aggressive solvents (HFIP and TFA) leads to a disruption of the amorphous phase of the elastomers, and decrease of ∆c_p_.

For the sample taken directly after polycondensation, and samples of soluble polymer fraction obtained by extraction (sol fraction) melting transition observable for insoluble fraction (T_m1_) splits into two, and two melting transitions can be observed. First melting transition (T_m2_) is a result of melting of poly (xylitol sebacate) blocks, and second melting transition (T_m3_) is a result of melting of poly (butylene sebacate) blocks. For samples after extraction, poly (butylene sebacate) blocks cannot crystalize properly when solvent evaporates, which leads to either decreased enthalpy (∆H_m3_) (for DMSO and HFIP), or the transition not being observable at all (for THF and TFA).

### 2.6. Hydrolytic and Enzymatic Degradation

**Figure 12:**
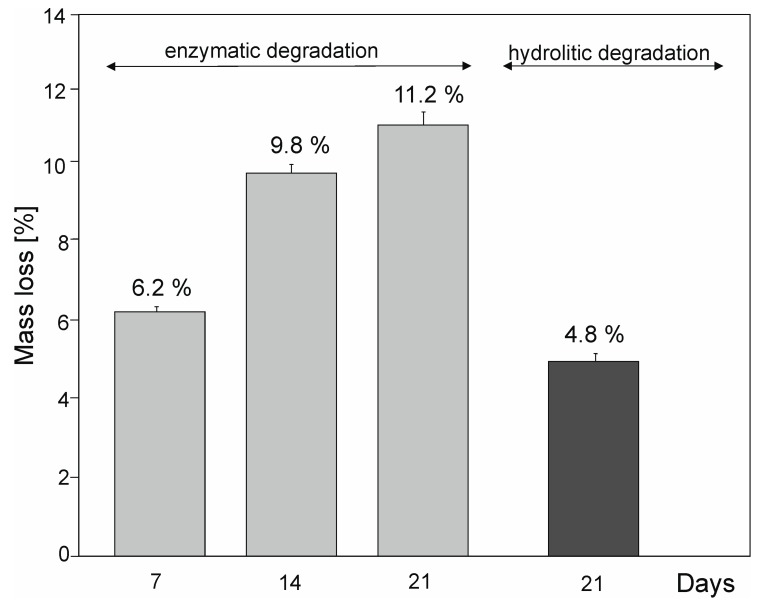
The enzymatic and hydrolytic degradation of PXBS.

Results of hydrolytic and enzymatic degradation are shown in Figure 12.

Rate of enzymatic degradation is faster than the rate of hydrolytic degradation. After 7 days of enzymatic degradation, the mass loss (6.2%) is higher than the mass loss (4.8%) of the sample after 21 days of hydrolytic degradation. We have compared the results with degradation times of commercially available biodegradable polyesters: PLLA-co-PGA, PGA, and PDS and concluded that our material is biodegradable [27].

## 3. Materials and Methods

### 3.1. Synthesis of PXBS

All reagents were purchased from Sigma-Aldrich (St. Louis, MO, USA). Xylitol-containing copolymer:poly(xylitol sebacate-co-butylene sebacate) was synthesized with a sebacic acid:butylene glycol:xylitol ratio of 2:1:1 (PXBS). The monomers were melted in a flask at a temperature above 100 °C under a blanket of N_2_. The esterification reaction was then carried out for 13.5 h at 150 °C in N_2_ atmosphere, catalyzed by Ti (OBu)_4_. The next step was a 3,5 h polycondensation reaction at 150 °C in a vacuum. The prepolymer was then cross-linked in a vacuum dryer at 100 °C in 100 mBar.

### 3.2. Extraction

Cross-linked polymer samples (10 g) were placed in a Soxhlet apparatus and subjected to extraction in boiling tetrahydrofuran (THF), dichloromethane (DCM), dimethylformamide (DMF), dimethyl sulfoxide (DMSO), trifluoroacetic acid (TFA), ethyl acetate (ETAC), methanol (MeOH), chloroform (CHCl_3_), 1,1,1,3,3,3-hexafluoro-2-propanol (HFIP), n-methylpyrrolidone (NMP) (100 cm^3^). Samples resided in the solvents for 3 h. After extraction, the samples were dried in a vacuum oven at 25 °C and then in a desiccator.

Two kinds of samples were acquired: samples of insoluble cross-linked polymer fraction left after extraction (gel fraction), and samples of soluble polymer fraction obtained by extraction (sol fraction).

### 3.3. Experimental Methods

#### 3.3.1. Swelling

Equilibrium swelling in tetrahydrofuran (THF), dichloromethane (DCM), dimethylformamide (DMF), dimethyl sulfoxide (DMSO), trifluoroacetic acid (TFA), ethyl acetate (ETAC), methanol (MeOH), chloroform (CHCl_3_), 1,1,1,3,3,3- hexafluoro-2-propanol (HFIP), and n-methylpyrrolidone (NMP) was measured by calculating the relative percentage increase in the mass of the cross-linked samples residing in the solvents for 72 h at 20 °C, in accordance with PN-EN 579: 2001 method. Each sample subjected to swelling was 10 g. The content of swelling [%] was calculated from formula (1) as the mean of three measurements:

Ε = (l_t_ −l_0_)/l_o_·100% (1)

where: l_t_—mass after time t = 72 h, l_0_—mass at time t = 0.

#### 3.3.2. Gel Fraction

Determination of the gel fraction of elastomer after cross-linking was made by the extraction method PN-EN 579:2001. Samples of the material after cross-linking (about 10 g) were placed in Schott type P2 crucible and subjected to extraction in boiling tetrahydrofuran (THF), dichloromethane (DCM), dimethylformamide (DMF), dimethyl sulfoxide (DMSO), trifluoroacetic acid (TFA), ethyl acetate (ETAC), methanol (MeOH), chloroform (CHCl_3_), 1,1,1,3,3,3- hexafluoro-2-propanol (HFIP), n-methylpyrrolidone (NMP) (100 cm^3^) for 3 h. After extraction, the samples were dried in a vacuum oven at 25 °C and then in a desiccator. Three determinations were made for each sample. The content of gel fraction was calculated from formula (2) as the mean of three measurements:

X = m_1_/m_0_∙100% (2)

where: m_1_—sample mass after extraction, m_0_—sample mass before extraction

#### 3.3.3. Fourier Transform Infrared Spectroscopy (FTIR)

Fourier transform infrared spectroscopy (ATR FTIR Alpha spectrometer, Bruker, Germany) was used to examine the chemical structure of the PXBS polymer. Sample of the polymer after cross-linking and samples of insoluble cross-linked polymer fraction left after extraction (gel fraction) were analyzed. Sample of polymer after polycondensation, and samples of soluble polymer fraction obtained by extraction (sol fraction) were also examined. FTIR transmittance spectra were recorded between 400 and 4000 cm^−1^, with a resolution of 2 cm^−1^, and 32 scans. Test results were developed using Omnic software.

#### 3.3.4. NMR Spectroscopy

Nuclear magnetic resonance spectroscopy (NMR) was used to analyze the chemical structure of PXBS copolymer, and xylitol used for the synthesis. Analysis of the polymer was performed on four polymer samples. One sample was taken directly after polycondensation, and the next three samples were obtained by performing extraction of the cross-linked polymer, using three different solvents—dimethyl sulfoxide (DMSO), methanol (MeOH), and tetrahydrofuran (THF). All polymer samples were dissolved in deuterated chloroform (CDCl_3_). Xylitol sample was dissolved in water. Measurements were performed on Bruker DPX 400 MHz. Results were analysed using MestReNova program.

#### 3.3.5. Differential Scanning Calorimetry (DSC).

Thermal properties were analyzed using differential scanning calorimetry (DSC) (Q 2500, TA Instruments apparatus, New Castle, USA). The measurement was carried out in a heating cycle in temperature range from −100 to 100 °C, and 10 °C/min heating rate in nitrogen atmosphere. Sample of polymer after cross-linking and samples of insoluble cross-linked polymer fraction left after extraction (gel fraction) were analyzed. Sample of polymer after polycondensation, and samples of soluble polymer fraction obtained by extraction (sol fraction) were also examined. Weight of the samples was 10–15 mg. Results were analyzed with TA Instruments Universal Analysis program.

#### 3.3.6. Mechanical Properties

Mechanical tests were carried out with an Instron 3366 instrument equipped with a 500 N load cell in accordance with standard PN-EN-ISO 527/1:1996 (crosshead speed of 100 mm/min, at 25 °C and 50% of relative humidity). Measurements were performed on a fully cross-linked polymer sample.

#### 3.3.7. Water Contact Angle

Water contact angle was measured using a KRUSS DSA100 digital goniometer. Static contact angle measurements were performed on the surface of degreased material by placing a 2 μL droplet of deionized water using the automatic dispenser of the goniometer. Contact angle was calculated using Kruss drop shape analysis software (DSA4). Measurements were performed on a fully cross-linked polymer sample.

#### 3.3.8. Hydrolytic Degradation

Hydrolytic degradation was carried out on previously sterilized 10 mm (0.5 g) polymer discs obtained from not-extracted, fully cross-linked material, for 21 days in phosphate-buffered saline (PBS) (Sigma Aldrich, Poznań, Poland) (pH range 7.1–7.2), in 37 °C. PBS solution was changed every 48 h. Sterilization was conducted in a laminar chamber for 15 min, using UV light. Samples were placed in a 24-well-plate. Each sample was covered with 1.5 mL of the solution. Samples after degradation were dried in a vacuum dryer in 25 °C and then weighed. Mass loss after 21 days was calculated using formula (3)

D = (m_0_ − m_1_)/m_0_∙100% (3)

where D—mass loss [%], m_0_—sample mass before degradation [g], m_1_—sample mass after degradation [g].

#### 3.3.9. Enzymatic Degradation

Enzymatic degradation was carried out on previously sterilized 10 mm (0.5 g) polymer discs obtained from not-extracted, fully cross-linked material for 21 days in solution of lipase (*Pseudomonas Cepacia*) (Sigma Aldrich, Poznań, Poland) (25 units/mL) in phosphate-buffered saline (PBS) (Sigma Aldrich, Poznań, Poland) (pH range 7.1–7.2). Lipase solution was changed every 48 h. Sterilization was conducted in a laminar chamber for 15 min, using UV light. Samples were placed in a 24-well-plate. Each sample was covered with 1.5 mL of the solution. Samples after degradation were dried in vacuum dryer in 25 °C and then weighed. Mass loss after 7, 14, and 21 days was calculated using formula (4)

D = (m_0_ − m_1_)/m_0_∙100% (4)

where D—mass loss [%], m_0_—sample mass before degradation [g], m_1_—sample mass after degradation [g].

## 4. Conclusions

In this paper we describe a biodegradable polymer which belongs to a recently developed group of xylitol-based materials.

It is characterized by good mechanical properties typical for the elastomer group. We have concluded that an exact determination of the polymer structure was necessary to better understand its behavior and properties. Because of the insolubility of cross-linked polymer, we have performed an extraction of this material using different solvents in order to obtain samples suitable for in-depth analysis. It has also allowed us to analyze how the polymer behaves after being subjected to different chemical environments.

NMR analysis has proven that this polymer has a structure consisting of poly (xylitol sebacate) and poly (butylene sebacate) blocks with dicarboxylic acid creating bonds between polymer chains during cross-linking process.

Furthermore, the structure was additionally confirmed by DSC analysis. Two characteristic melting temperatures from two different blocks were observed. Polymer biodegradability was also confirmed.

Additionally, it is worth noting that elastomers synthesized using butylene glycol as third monomer have better mechanical properties than materials synthesized using only sugar-alcohol and dicarboxylic acid, described in the literature.

## Figures and Tables

**Table 1:** Thermal properties of PXBS sample after cross-linking and samples of insoluble cross-linked PXBS fraction left after extraction (PXBS_GEL) (**A**) and thermal properties of the PXBS sample taken after polycondensation (PXBS_co) and samples of soluble PXBS fraction obtained by extraction (PXBS_SOL) (**B**), first heating.

Sample/Material	PXBS_GEL				Sample/Material	PXBS_SOL			
	T_g1_ [°C]	∆c_p_ [J/g°C]	T_m1_ [°C]	∆H_m1_ [J/g]		T_m2_ [°C]	∆H_m2_ [J/g]	T_m3_ [°C]	∆H_m3_ [J/g]
**PXBS_Cross-Linked**	−29.9	0.412	16.8	26.3	PXBS_co	19.1	26.4	44.8	50.9
PXBS_THF	−28.3	0.550	11.1	9.1	PXBS_THF	16.5	44.2	n.o	n.o
PXBS_DMSO	−31.7	0.439	14.7	28.2	PXBS_DMSO	20.4	33.3	47.9	18.6
PXBS_HFIP	−25.8	0.342	18.4; 41.8	26.4; 1.6	PXBS_HFIP	20.1	29.8	41.2	14.8
PXBS_TFA	−32.3	0.143	9.1; 20.8	40.3	PXBS_TFA	11.1; 25.2	19.8	n.o	n.o.

n.o.—not observed, where: T_g1_-glass transition temperatures; ∆c_p_-change of the heat capacity at glass transition, T_m1_, T_m2_, T_m3_-melting temperature; ΔH_m1,_ ΔH_m2,_ ΔH_m3_-enthalpy of melting at T_m1_, T_m2_, T_m3_.

## Supplementary Materials

The Supplementary Materials are available online. DSC results Table S1.
