# Supplementary material for: Structure and Properties of Biodegradable Poly (Xylitol Sebacate-Co-Butylene Sebacate) Copolyester
Source: Molecules. 2020 Mar 28;25(7):1541. doi: 10.3390/molecules25071541 (PMC7180773; doi:10.3390/molecules25071541)
Supplement: Supplementary file 1 [file molecules-25-01541-s001.pdf]

Table S1

Table S1. Thermal properties of PXBS sample after cross-linking and samples of insoluble cross-linked PXBS fraction left after extraction (PXBS_GEL) (A) and thermal properties of the PXBS sample taken after polycondensation (PXBS_co) and samples of soluble PXBS fraction obtained by extraction (PXBS_SOL) (B), first heating

Sample /Material	PXBS_GEL				Sample /Material	PXBS_SOL			
	T_{g1} [°C]	Δc_p [J/g°C]	T_{m1} [°C]	ΔH_{m1} [J/g]		T_{m2} [°C]	ΔH_{m2} [J/g]	T_{m3} [°C]	ΔH_{m3} [J/g]
PXBS_cross-linked	-29.9	0.412	16.8	26.3	PXBS_co	19.1	26.4	44.8	50.9
PXBS_THF	-28.3	0.550	11.1	9.1	PXBS_THF	16.5	44.2	n.o	n.o
PXBS_DMSO	-31.7	0.439	14.7	28.2	PXBS_DMSO	20.4	33.3	47.9	18.6
PXBS_HFIP	-25.8	0.342	18.4;41.8	26.4;1.6	PXBS_HFIP	20.1	29.8	41.2	14.8
PXBS_TFA	-32.3	0.143	9.1;20.8	40.3	PXBS_TFA	11.1;25.2	19.8	n.o	n.o.

n.o. – not observed

where: T_{g1} *- glass transition temperatures;* Δc_p *- change of the heat capacity at glass transition,* T_{m1}, T_{m2}, T_{m3}, *- melting temperature;* ΔH_{m1}, ΔH_{m2}, ΔH_{m3}, *- heat of melting at* T_{m1}, T_{m2}, T_{m3}.
